# p53 target ANKRA2 cooperates with RFX7 to regulate tumor suppressor genes

**DOI:** 10.1038/s41420-024-02149-2

**Published:** 2024-08-24

**Authors:** Katjana Schwab, Konstantin Riege, Luis Coronel, Clara Stanko, Silke Förste, Steve Hoffmann, Martin Fischer

**Affiliations:** 1 Computational Biology Group, Leibniz Institute on Aging—Fritz Lipmann Institute (FLI), Jena, Germany; 2 Klinik für Innere Medizin II, Jena University Hospital, Comprehensive Cancer Center Central Germany, Jena, Germany; 3 Institute of Molecular Cell Biology, Center for Molecular Biomedicine Jena (CMB), Jena University Hospital, Jena, Germany

**Keywords:** Gene regulation, Transcriptomics, Proteomics, Stress signalling

## Abstract

The transcription factor regulatory factor X 7 (RFX7) has been identified as a tumor suppressor that is recurrently mutated in lymphoid cancers and appears to be dysregulated in many other cancers. RFX7 is activated by the well-known tumor suppressor p53 and regulates several other known tumor suppressor genes. However, what other factors regulate RFX7 and its target genes remains unclear. Here, reporter gene assays were used to identify that RFX7 regulates the tumor suppressor gene *PDCD4* through direct interaction with its X-box promoter motif. We utilized mass spectrometry to identify factors that bind to DNA together with RFX7. In addition to RFX7, we also identified RFX5, RFXAP, RFXANK, and ANKRA2 that bind to the X-box motif in the *PDCD4* promoter. We demonstrate that *ANKRA2* is a bona fide direct p53 target gene. We used transcriptome analyses in two cell systems to identify genes regulated by ANKRA2, its sibling RFXANK, and RFX7. These results revealed that ANKRA2 functions as a critical cofactor of RFX7, whereas RFXANK regulates largely distinct gene sets.

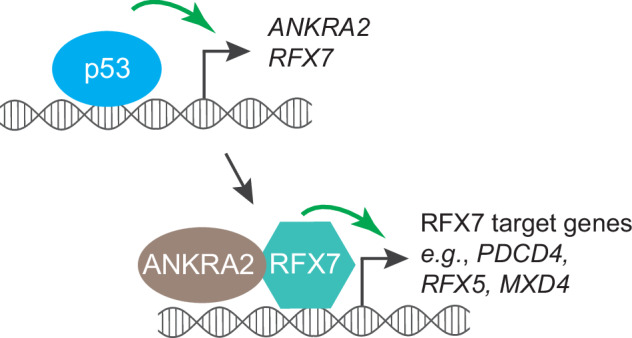

## Introduction

RFX7 has recently emerged as a tumor suppressor that is recurrently mutated in hematopoietic cancers [1]. For example, *RFX7* mutations have been implied to be cancer drivers in Burkitt lymphoma [2, 3] and chronic lymphocytic leukemia [4]. In strong support of its tumor suppressor function, loss of *RFX7* accelerated B cell lymphomagenesis in mouse models [5]. In addition, RFX7 appears to be deactivated in many cancers in which it is not mutated [6], making RFX7 reactivation an intriguing target for cancer therapy. Besides cancer, *RFX7* has been implicated in organismal development [7], neurological disorders [8–11], metabolic disorders [12], and immune cell maintenance [13].

The regulatory factor X (RFX) transcription factor family is evolutionarily conserved in the eukaryotic kingdom [14, 15]. In humans, the RFX family consists of eight members, all of which possess a conserved winged-helix DNA-binding domain (DBD) through which they can bind *cis*-regulatory X-box DNA motifs [16, 17]. Most *RFX* genes show cell type-specific expression, but *RFX1*, *RFX5*, and *RFX7* are expressed in essentially all tissues [17, 18]. *RFX5* is the phylogenetically closest sibling of *RFX7* and the most extensively studied member of the RFX family. RFX5 regulates major histocompatibility complex (MHC) class II genes. Together with RFXANK and RFXAP, RFX5 can form the RFX complex that recruits the transcriptional coactivator CIITA to promote MHC class II gene expression [19]. Unlike RFX5, its sibling RFX7 is poorly understood.

Recently, we found that the tumor suppressor p53 activates RFX7 upon p53-activating stress signals [6, 20]. We have also identified genes directly and indirectly regulated by RFX7 genome-wide, including genes involved in neuronal processes, metabolic regulation, and tumor suppressor genes such as *PDCD4*, *DDIT4*, and *PIK3IP1* [6, 21, 22]. While most RFX family members bind to an X-box motif containing two palindromic half-sites of the sequence 5′-GTTGCCA-TGGCAAC-3′, RFX5 and RFX7 bind to a non-palindromic variant with the sequence 5′-CCTAGCAACA-3′ [6]. In addition, the binding sites of both RFX7 and RFX5 are often located near CCAAT-boxes that recruit the histone-like transcription factor NF-Y [6, 19], which consists of the three subunits NF-YA, NF-YB, and NF-YC [23]. Despite these similarities, RFX7 binds to only a small fraction of RFX5 binding sites [6], suggesting that other factors influence their ability to bind to DNA. RFX7 may be an attractive target for cancer therapy because it is mutated in a relatively small number of cancers but appears to be inactivated in many cancers [6]. To identify therapeutically exploitable targets in the RFX7 signaling pathway, it is critical to better understand the factors that affect RFX7 activity.

Here, we dissected the RFX7-dependent regulation of the RFX7 target genes *PDCD4*, *RFX5*, and *MXD4*. We demonstrate the importance of the X-box promoter motif and used mass spectrometry for unbiased identification of X-box binding factors. Furthermore, we assessed the significance of the identified factors in RFX7-mediated gene regulation. Collectively, our results identify the p53 target ANKRA2 as a critical cofactor of RFX7.

## Results

### RFX7 functions through X-box DNA motifs

**Fig. 1:**
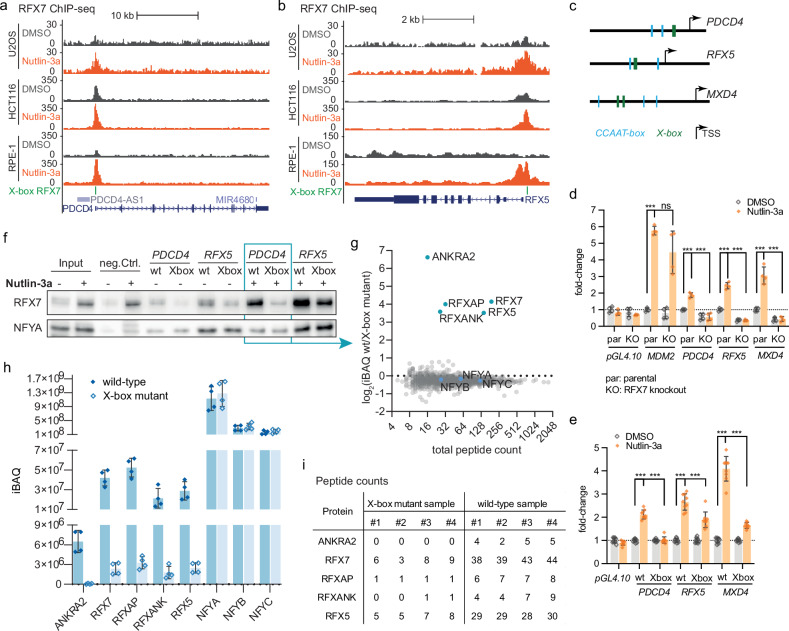
Function and protein binding mediated by X-box promoter elements. UCSC genome browser images displaying RFX7 ChIP-seq signals and predicted X-boxes [6] at the (**a**) *PDCD4* and (**b**) *RFX5* gene loci. **c** Schematics of the *PDCD4*, *RFX5*, and *MXD4* promoters that were cloned. We identified X-box (green) and CCAAT-box motifs (blue) in both promoters. The arrows indicate the TSS of the canonical transcript. The schematics are drawn to scale. **d** Dual-luciferase assay of *MDM2*, *PDCD4*, *RFX5*, and *MXD4* promoter activity in DMSO control and Nutlin-3a-treated U2OS parental (par) and RFX7 knockout (KO) cells and (**e**) *PDCD4*, *RFX5*, and *MXD4* promoter activity of wild-type (wt) or X-box mutant (Xbox) in DMSO control and Nutlin-3a-treated U2OS cells. An empty pGL4.10 vector served as a negative control. Normalized to DMSO control. Mean and standard deviation are displayed. Statistical significance was obtained through a two-sided unpaired t-test, *n* = 4 or *n* = 9 biological replicates. **f** Western blot data from DNA affinity purification eluates using biotinylated *PDCD4* and *RFX5* wild-type (wt) and X-box deleted (Xbox) promoter probes with eluates from DMSO and Nutlin-3a-treated U2OS cells. NF-YA binding served as a loading control. Uncropped western blot images are shown in Supplementary Fig. 2A. **g** Protein enrichment identified through mass spectrometry analysis of *PDCD4* wild-type (wt) and X-box mutant DNA affinity purification eluates using Nutlin-3a treated U2OS cells. **h** iBAQ protein expression values from mass spectrometry analysis of DNA affinity purification eluates from *PDCD4* wild-type and X-box mutant probes incubated with cell lysates from Nutlin-3a treated U2OS cells. Mean and standard deviation are displayed. ANKRA2, RFX7, RFXAP, RFXANK, and RFX5 were significantly enriched at *PDCD4* wild-type samples. The components of the CCAAT-box binding NF-Y complex, NFYA, NFYB, and NFYC, served as control. **i** Peptide counts for highly enriched binding proteins from the mass spectrometry analysis; complementary to (**g**, **h**).

Previously, we identified the p53-RFX7 signaling pathway and charted direct RFX7 target genes. De novo DNA recognition motif searches identified X-box and CCAAT-box sequences enriched under ChIP-seq-derived RFX7 binding sites [6]. To test for the importance of the X-box sequence for RFX7 DNA binding and target gene regulation, we cloned the proximal promoters of *PDCD4*, *RFX5*, and *MXD4* (Fig. 1a–c and Supplementary Fig. 1a), which represent three direct RFX7 target genes that we previously identified [6]. We chose *PDCD4*, *RFX5*, and *MXD4* because they display RFX7 binding just proximal to their transcription start site (TSS) (Fig. 1a, b), allowing for a straightforward assay design. Consistent with previous findings [6], ChIP-seq data from three cell lines indicate increased RFX7 occupancy at both genes upon treatment with the MDM2 inhibitor and p53 activator Nutlin-3a (Fig. 1a, b and Supplementary Fig. 1a). In addition to a previously identified X-box [6], we identified CCAAT-boxes in each promoter region, potentially recruiting the transcription factor NF-Y (Fig. 1c).

We tested the activity of the *PDCD4*, *RFX5*, and *MXD4* promoters in dual-luciferase assays for their response to p53 activation. We treated U2OS cells with the MDM2 inhibitor Nutlin-3a to activate p53 [24]. In addition to parental U2OS cells, we used U2OS cells carrying an RFX7 knockout [22]. The *MDM2* promoter served as a positive control for an RFX7-independent response to p53 activation. While the *MDM2* promoter conferred luciferase activity upon Nutlin-3a treatment in both parental and RFX7^−/−^ U2OS cells, the *PDCD4*, *RFX5*, and *MXD4* promoters were activated only in the parental U2OS cells (Fig. 1d). These results provide evidence that the p53-RFX7 signaling pathway regulates the *PDCD4*, *RFX5*, and *MXD4* promoters.

To test for the function of the predicted X-box motifs, we deleted the corresponding sequences from the *PDCD4*, *RFX5*, and *MXD4* promoters. Dual-luciferase assays showed that activation of the *PDCD4*, *RFX5*, and *MXD4* promoters upon Nutlin-3a treatment was lost when the X-box motif was deleted (Fig. 1e). These results underline that the X-box sequences are critical for p53-RFX7-mediated regulation of the promoters. Next, we incubated wild-type and X-box mutant *PDCD4*, *RFX5*, and *MXD4* promoter probes with nuclear extracts from Nutlin-3a and DMSO control-treated U2OS cells. We performed DNA-affinity purification followed by immunoblot analysis to test for RFX7 binding to the *PDCD4* and *RFX5* promoters. Consistent with the ChIP-seq data (Fig. 1a, b and Supplementary Fig. 1a), RFX7 binding to the wild-type *PDCD4*, *RFX5*, and *MXD4* promoters increased upon Nutlin-3a treatment. Notably, the deletion of the X-box substantially reduced RFX7 binding to the promoters. In the case of *PDCD4* and *MXD4*, RFX7 binding was reduced to background control levels upon X-box deletion. At the same time, NF-YA binding was unaffected and served as a loading control (Fig. 1f and Supplementary Fig. 1a). The residual activation of the *RFX5* X-box mutant promoter upon Nutlin-3a treatment (Fig. 1e) and the reduced but persistent RFX7 binding (Fig. 1f) suggested that the *RFX5* promoter may contain another unidentified RFX7 binding sequence. Therefore, we selected the *PDCD4* promoter for a subsequent high-throughput analysis of protein binding (see next section).

Taken together, the results show that the p53-RFX7 signaling pathway activates the proximal promoters of *PDCD4*, *RFX5*, and *MXD4*. Furthermore, RFX7 requires the predicted X-box motifs to bind and fully activate the promoters.

### Protein binding to the *PDCD4* X-box promoter element

To identify factors that might act together with RFX7 at X-box promoter motifs, we examined protein binding to the X-box motif of the *PDCD4* promoter by mass spectrometry. We compared DNA-affinity purification eluates from wild-type and X-box mutant *PDCD4* promoter probes incubated with Nutlin-3a-treated cell extracts. Mass spectrometry analysis identified ANKRA2, RFX7, RFXAP, RFXANK, and RFX5 as significantly enriched in wild-type compared to X-box mutant promoter samples. In contrast, other RFX family members, including the ubiquitously expressed RFX1, were not identified as enriched (Fig. 1g–i). The NF-Y subunits NF-YA, NF-YB, and NF-YC bound to the wild-type and X-box mutant *PDCD4* promoters, presumably through the intact CCAAT-boxes. (Fig. 1g, i).

Of note, RFX5, RFXAP, and RFXANK are known to form the RFX complex that regulates MHC class II gene expression [19], and there are conflicting reports as to whether its sibling ANKRA2 can replace RFXANK to regulate these genes [25, 26]. In addition to their ability to form a complex with RFX5, the paralogous proteins RFXANK and ANKRA2 have also been shown to bind to RFX7 [27, 28]. While RFXANK preferentially binds to RFX5, its sibling ANKRA2 appears to have a higher binding affinity for RFX7 [27]. We have previously found that depletion of RFX5 does not affect the activation of RFX7 target genes [6], suggesting that the RFX complex does not affect RFX7. Given their ability to bind to RFX7, we investigated the role of ANKRA2 and RFXANK in RFX7-dependent gene regulation.

### *ANKRA2* is a direct p53 target

**Fig. 2:**
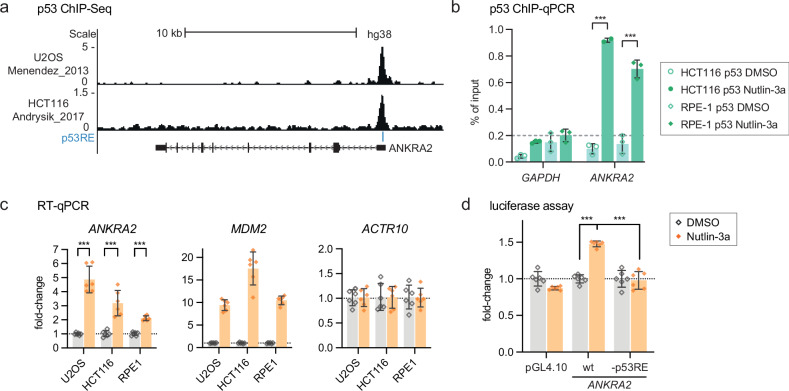
*ANKRA2* is a direct p53 target gene. **a** UCSC genome browser image displaying the *ANKRA2* gene locus. Publicly available p53 ChIP-seq signals from Nutlin-3a treated U2OS and HCT116 cells [33] and predicted p53 response elements (p53RE) [34] are displayed. **b** ChIP-qPCR data of p53 binding to the promoters of *ANKRA2* and *GAPDH* negative control. Mean and standard deviation are displayed. Statistical significance was obtained through a two-sided unpaired t-test, n = 3 technical replicates. **c** RT-qPCR data of *ANKRA2* in U2OS, HCT116, and RPE1 cells. Normalized to *ACTR10* negative control and DMSO samples. *MDM2* serves as a positive control for p53 induction by Nutlin-3a. Mean and standard deviation are displayed. Statistical significance was obtained through a two-sided unpaired *t*-test, *n* = 6 replicates (two biological with three technical each). **d** Dual-luciferase assay of wild-type (wt) or p53RE deleted (-p53RE) *ANKRA2* promoter activity in DMSO control and Nutlin-3a-treated U2OS cells. An empty pGL4.10 vector served as a negative control. Normalized to DMSO control. Mean and standard deviation are displayed. Statistical significance was obtained through a two-sided unpaired t-test, *n* = 6 biological replicates.

Interestingly, *ANKRA2* has been identified as a potential p53 target gene [29–32], whereas *RFXANK* expression does not appear to be induced by p53 signaling [33]. Publicly available data indicate that p53 binds near the TSS of *ANKRA2* (Fig. 2a). In agreement with previous data, our ChIP-qPCR data showed inducible p53 binding to the *ANKRA2* promoter (Fig. 2b), and our RT-qPCR data showed that *ANKRA2* mRNA levels increased significantly in response to Nutlin-3a treatment (Fig. 2c). We next sought to test whether *ANKRA2* was upregulated through the p53 binding site in its promoter. We cloned the *ANKRA2* promoter region and deleted a p53 response element (p53RE) that had been predicted by motif analysis [34]. Results from dual-luciferase assays showed that the *ANKRA2* promoter mediated transcriptional activation upon Nutlin-3a treatment and that this activation was lost when the p53RE was deleted (Fig. 2d). Collectively, these results demonstrate that *ANKRA2* is a direct target gene of p53.

### ANKRA2 is required for p53-RFX7 signaling

**Fig. 3:**
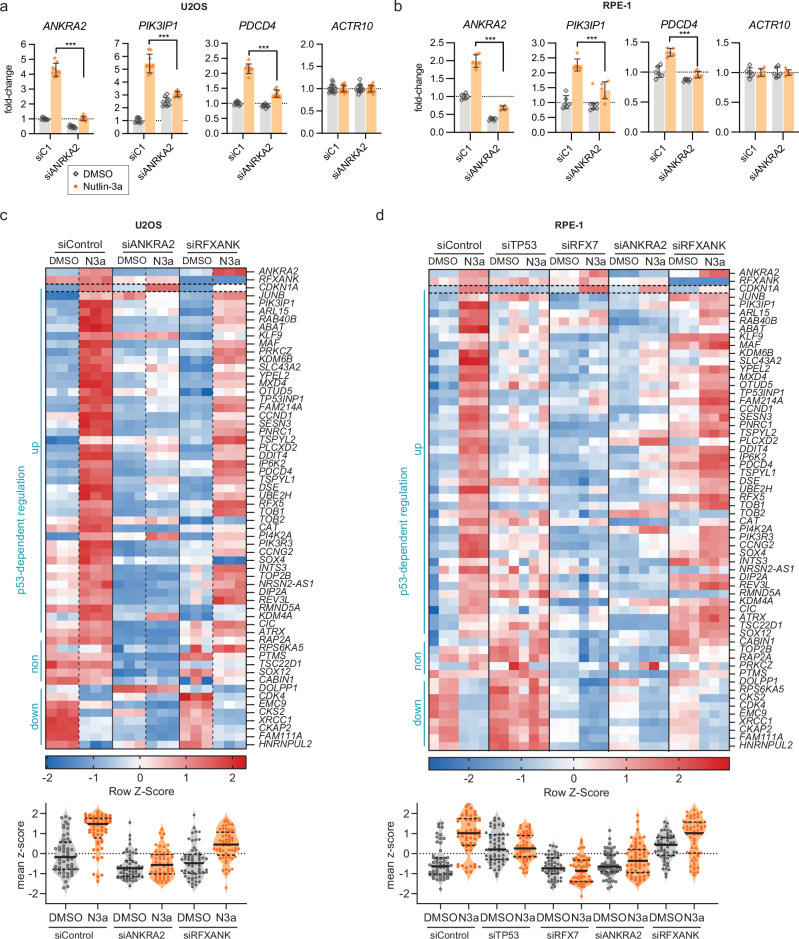
ANKRA2 is required for p53-RFX7 signaling. RT-qPCR data from (**a**) U2OS and (**b**) RPE-1 cells depleted for ANKRA2 and treated with DMSO or Nutlin-3a. Normalized to *ACTR10* negative control and siControl DMSO. Mean and standard deviation are displayed. Statistical significance was obtained through a two-sided unpaired *t*-test, *n* = 9 (U2OS) or *n* = 6 (RPE-1). Heatmap of RNA-seq data for 57 direct RFX7 target genes [6] from (**c**) U2OS and (**d**) RPE-1 cells depleted for ANKRA2 and treated with DMSO or Nutlin-3a. *ANKRA2*, *RFXANK*, and the direct p53 target *CDKN1A* serve as controls. Genes that were significantly regulated by p53 (Nutlin-3a vs DMSO control of siControl transfected cells; FDR < 0.05) are indicated as up- (log2FC > 0.25) and downregulated (log2FC < −0.25).

Given that ANKRA2 is a direct p53 target (Fig. 2), similar to RFX7 [6], and given their common binding to the X-box motif of the *PDCD4* promoter (Fig. 1g–i), we tested whether ANKRA2 affects the activation of the direct RFX7 targets upon p53 signaling. We used siRNA to knock down *ANKRA2* and assessed its effect on the expression of the direct RFX7 targets *PDCD4* and *PIK3IP1* [6] in response to Nutlin-3a treatment in U2OS and RPE-1 cells. RT-qPCR analysis showed that *ANKRA2* knockdown significantly impaired the Nutlin-3a-induced activation of *PDCD4* and *PIK3IP1* (Fig. 3a, b).

To gain a more comprehensive insight into the transcriptional effects of ANKRA2 on RFX7 targets, we performed RNA-seq analysis of *ANKRA2*-depleted U2OS and RPE-1 cells. In addition, we evaluated the effect of *RFXANK* knockdown in these cells. We also included *RFX7* and *TP53* (p53) knockdown in RPE-1 cells as an additional control. Knockdown of *ANKRA2* impaired the Nutlin-3a-induced expression of most of the 57 direct RFX7 target genes we previously identified (Fig. 3c, d). Like RFX7 depletion, ANKRA2 knockdown had essentially no effect on the induction of the direct p53 target *CDKN1A* upon Nutlin-3a treatment, confirming the function of ANKRA2 downstream of p53. In contrast to *ANKRA2* and *RFX7* depletion, *RFXANK* knockdown only slightly reduced the Nutlin-3a-induced expression of direct RFX7 targets in U2OS cells. In contrast, *RFXANK* knockdown slightly increased the expression of these genes in DMSO control-treated RPE-1 cells, whereas it essentially did not affect the Nutlin-3a-induced expression of these genes in RPE-1 cells. While the knockdown of RFXANK had small effects on the direct RFX7 target genes at large, it only affected individual genes, and the effect was inconsistent between the two cell systems (Fig. 3c, d). Thus, our data suggest that the direct p53 target ANKRA2 is an important cofactor of RFX7, while RFXANK does not appear to play a general role in this signaling axis.

**Fig. 4:**
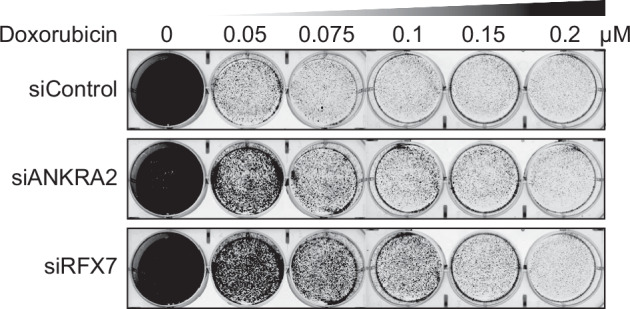
ANKRA2 depletion desensitizes cells to Doxorubicin treatment. Clonogenic assay of HCT116 cells transfected with indicated siRNAs 5 days post-recovery from a treatment with different concentrations of Doxorubicin for 24 h.

We have previously shown that RFX7 can sensitize cells, such as HCT116 colorectal cancer cells, to low concentrations of the chemotherapeutic drug Doxorubicin [6]. Given the transcriptional effect of ANKRA2 on the p53-RFX7 signaling, we tested whether ANKRA2 also influences the cell fate upon Doxorubicin treatment. Indeed, a clonogenic assay following ANKRA2 depletion shows a desensitization of HCT116 cells to low concentrations of Doxorubicin, although to a slightly lesser extent than depletion of RFX7 itself (Fig. 4).

## Discussion

The evolutionarily well-conserved transcription factor RFX7 was identified in 2008 [18], and it was only recently that RFX7 was identified as a tumor suppressor when a larger number of whole-genome sequencing analyses of cancer samples became available [2–4]. In conjunction with its role within the p53 network [6], these findings highlight RFX7 as a potentially potent tumor suppressor that may be of therapeutic interest because it is mutated in a relatively small number of cancers and thus has the potential to be activated. We recently revealed that RFX7 controls the expression of several known tumor suppressors, further substantiating its tumor suppressor function [6, 22]. However, it is unclear which pathways other than p53 use RFX7 and which cofactors RFX7 requires for target gene activation.

Our results establish ANKRA2 as an important cofactor of RFX7. We identified that ANKRA2 binds to the same X-box motif in the *PDCD4* promoter as RFX7 (Fig. 1g–i) and that ANKRA2 is required for the activation of RFX7 target genes within the p53-RFX7 signaling axis (Fig. 3). Based on these results, we propose a model in which RFX7 cooperates with ANKRA2 to upregulate its target genes. Importantly, we also identified *ANKRA2* as a direct target gene of p53 (Fig. 2), similar to *RFX7* [6]. Notably, both *ANKRA2* and *RFX7* are activated by various p53-inducing stimuli (see data at www.targetgenereg.org) and can be considered to be part of the “core program” of p53. Ultimately, the orchestrated modulation of ANKRA2 and RFX7 expression could enable p53 to fine-tune the signaling via its RFX7 axis.

Although our results establish that ANKRA2 functions as an important cofactor of RFX7, we find that the loss of RFX7 has a more pronounced effect than the loss of ANKRA2 (Figs. 3d and 4). This suggests that either ANKRA2 is not a rate-limiting factor in the RFX7:ANKRA2 interaction or that RFX7 can retain partial activity without ANKRA2. The greater importance of RFX7 in this interaction could explain why only *RFX7* mutations, but not *ANKRA2* mutations, have been associated with driver events in hematopoietic cancers [2–4]. However, non-synonymous mutations in *ANKRA2* have been identified as significantly enriched in oral squamous cell carcinoma [35], underscoring a potential tumor suppressor role for ANKRA2.

In addition to ANKRA2, we identified RFX5, RFXAP, and RFXANK, all components of the RFX complex, that bind to the X-box motif in the *PDCD4* promoter (Fig. 1g–i). Consistent with our previous findings that RFX5 does not affect RFX7 target genes [6], we found that its complex partner, RFXANK, also does not appear to have a general role in regulating RFX7 targets (Fig. 3c, d). We conclude that the RFX complex has little effect on the expression of RFX7 targets and that ANKRA2, but not its sibling RFXANK, is a functionally important RFX7 binding partner. However, it appears biophysically unlikely that the RFX complex and RFX7:ANKRA2 can occupy the same X-box motif at the same time. Therefore, future research needs to determine how they coordinate binding to X-box promoter motifs.

Overall, our findings will help to stimulate further research into the regulation of RFX7:ANKRA2, which may lead to new therapeutic approaches.

## Materials and methods

### Cell culture, drug treatment, and transfection

U2OS and HCT116 cells (ATCC, Manassas, Virginia, USA) were grown in high glucose Dulbecco’s modified Eagle’s media (DMEM) with pyruvate (Thermo Fisher Scientific, Darmstadt, Germany). RPE-1 hTERT cells (ATCC) were cultured in DMEM:F12 media (Thermo Fisher Scientific). Culture media were supplemented with 10% fetal bovine serum (FBS; Thermo Fisher Scientific) and penicillin/streptomycin (Thermo Fisher Scientific). Cell lines were tested twice a year for *Mycoplasma* contamination using the LookOut Detection Kit (Sigma) and all tests were negative.

Cells were treated with DMSO (0.1%; Carl Roth, Karlsruhe, Germany) or Nutlin-3a (10 µM; Sigma Aldrich, Darmstadt, Germany) for 24 h. For knockdown experiments, cells were seeded in 6-well plates or 6 cm dishes and reverse transfected with 10 nM Silencer Select siRNAs (Thermo Fisher Scientific) using RNAiMAX (Thermo Fisher Scientific) and Opti-MEM (Thermo Fisher Scientific) following the manufacturer protocol.

### Plasmids and DNA probes

The human *PDCD4*, *RFX5*, *MXD4*, *MDM2*, and *ANKRA2* promoters were amplified from genomic DNA and ligated in the pGL4.10 vector (Promega, Madison, WI, USA). Mutations were introduced using the QuikChange site-directed mutagenesis protocol (Agilent Technologies, Santa Clara, CA, USA). DNA probes for affinity purification with the same sequences were obtained by PCR using a biotinylated primer for labeling the 3’-end (Thermo Fisher Scientific). Primer sequences are listed in Supplementary Table 1.

### Dual-luciferase reporter assay

U2OS cells were plated in 24-well plates (40,000 cells per well). Cells were transfected with 250 ng of *Firefly* luciferase reporter plasmid (pGL4.10) and 50 ng of *Renilla* luciferase plasmid (pGL4.70; Promega) using Lipofectamine 3000 (Thermo Fisher Scientific). Cells were cultured overnight before treatment with Nutlin-3a and DMSO control for 24 h. Cells were collected and luciferase activity was measured using the Dual Luciferase Assay Kit (Promega) and a GloMax 20/20 luminometer (Promega) following the manufacturer's instructions.

### DNA affinity purification

U2OS cells were treated with DMSO or Nutlin-3a. DNA affinity purification was performed similarly as described previously [36, 37]. Cells were washed with PBS, scraped, and centrifuged at 1000 g for 10 min. To isolate nuclei, cells were incubated in buffer A (10 mM HEPES–KOH, pH 7.9, 1.5 mM MgCl2, complete EDTA-free protease inhibitors, 10 mM KCl, 0.1 mM DTT) on ice for 15 min and centrifuged at 4000 × *g* for 10 min. Nuclei were incubated in buffer C (20 mM HEPES/KOH (pH 7.9), 1.5 mm MgCl2, 0.2 mm EDTA, 450 mM NaCl, 0.1 mM DTT, complete EDTA-free protease and phosphatase inhibitors) on ice for 15 min. Using a syringe with a 23 gauge needle 10 times, the nuclei were mechanically disrupted. Extracts were centrifuged at 20,000 × *g* and 4 °C to remove debris. Then, NaCl concentration was adjusted to 150 mM by dilution with buffer C (without NaCl). Protein concentration was determined by Pierce 660 nm assay (Thermo Fisher Scientific). Salmon sperm DNA (Sigma Aldrich) was added at a final concentration of 100 µg/ml and incubated at room temperature for 15 min. One microgram of biotinylated DNA probe was incubated with 1 mg protein extracts for 30 min at room temperature. 50 µl of magnetic streptavidin bead suspension (Miltenyi, Bergisch Gladbach, Germany) was added and incubated for an additional 5 min. Proteins binding to the probes were isolated using the μMACS Streptavidin Kit (Miltenyi). Washing was performed five times with 200 µl buffer C supplemented with 150 mM NaCl. Proteins were eluted in two steps with 20 µl and 60 µl elution buffer at 95 °C. Twenty microliters of the eluates and 15 µg nuclear extract (input) were used for SDS–PAGE followed by western blot. For Supplementary Fig. 1B, whole cell lysates were used. Cells were lysed using IP lysis buffer (25 mM Tris-HCl (pH 7.5), 150 mM NaCl, 1 mM EDTA, 1% NP-40, complete EDTA-free protease inhibitors, phosSTOP phosphatase inhibitor) on ice for 15 min. Nuclei were mechanically disrupted using a syringe with a 23 gauge needle 10 times. The extracts were then centrifuged at 20,000 g at 4 °C to remove debris. Protein concentration was determined by the BCA assay (Thermo Fisher Scientific). One microgram of biotinylated DNA probe was incubated with 1 mg of protein extracts at 4 °C for 60 min. Subsequently, 20 µL of magnetic streptavidin T1 bead suspension (Thermo Fisher Scientific) was added, followed by incubation at 4 °C for 15 min. The beads were washed four times with 150 µL of ice-cold IP wash buffer (25 mM Tris-HCl (pH 7.5), 150 mM NaCl, 1 mM EDTA, 0.1% NP-40). Proteins were eluted in 30 µL of elution buffer at 95 °C for 5 min. Ten microliters of the eluates and 5 µg of cell lysate were subjected to SDS–PAGE followed by western blot.

### Immunoblot analysis

Proteins were separated in a Mini-Protean TGX Stain-Free Precast 4–15% Gel (Bio-Rad) using Tris/Glycine/SDS running buffer (Bio-Rad). Proteins were transferred to a 0.2 µm polyvinylidene difluoride (PVDF) transfer membrane using a Mini Trans-Blot Cell (Bio-Rad) in a Mini-Protean Tetra Cell (Bio-Rad). Following antibody incubation, membranes were developed using Clarity Max ECL (Bio-Rad) and a ChemiDoc MP imaging system (Bio-Rad). Antibodies and their working concentrations are listed in Supplementary Table 1. All ANKRA2 antibodies we tested failed to detect endogenous ANKRA2.

### Proteomics

Four biological replicates of *PDCD4* wild-type and Xbox mutant promoter DNA affinity purification eluates from Nutlin-3a treated U2OS cells were used for mass spectrometry (MS) analysis. DNA-bound proteins were directly eluted with 10x lysis and reduction buffer (final concentration: 100 mM HEPES pH 8, 50 mM DTT, 2% SDS) and snap-frozen until processing. On preparation for MS, samples were thawed and sonicated (Bioruptor Plus, Diagenode) for 10 cycles (30 sec ON/60 sec OFF) at high setting, at 20 °C, followed by boiling at 95 °C for 5 min. Reduction was followed by alkylation with 20 mM iodoacetamide (IAA, final concentration 15 mM) for 30 min at room temperature in the dark. Proteins were precipitated overnight at −20 °C after addition of 8× volume of ice-cold acetone. 10 µg of protein were taken for further digestion. The following day, the samples were centrifuged at 20,800 × *g* for 30 min at 4 °C and the supernatant carefully removed (5810 R, Eppendorf, Hamburg, Germany). Pellets were washed twice with 300 µL ice-cold 80% (v/v) acetone in water, then centrifuged at 20800x g at 4 °C for 10 min. After removing the acetone, pellets were air-dried before addition of 25 µL of digestion buffer (1 M Guanidine, 100 mM HEPES, pH 8). Samples were resuspended with sonication as explained above, then LysC (Wako Chemicals, Richmond, VA, USA) was added at 1:100 (w/w) enzyme:protein ratio, and digestion proceeded for 4 h at 37 °C under shaking (1000 rpm for 1 h, then 650 rpm). Samples were then diluted 1:1 with MilliQ water and trypsin (Promega) was added at 1:100 (w/w) enzyme:protein ratio. Samples were further digested overnight at 37 °C under shaking (650 rpm). The day after, digests were acidified by the addition of TFA to a final concentration of 10% (v/v), heated at 37 °C and then desalted with Waters Oasis® HLB µElution Plate 30 µm (Waters Corporation, Milford, MA, USA) under a soft vacuum following the manufacturer instruction. Briefly, the columns were conditioned with 3 × 100 µL solvent B (80% (v/v) acetonitrile; 0.05% (v/v) formic acid) and equilibrated with 3 × 100 µL solvent A (0.05% (v/v) formic acid in Milli-Q water). The samples were loaded, washed three times with 100 µL solvent A, and then eluted into 0.2 mL PCR tubes with 50 µL solvent B. The eluates were dried down using a speed vacuum centrifuge (Concentrator Plus, Eppendorf) and redissolved at a concentration of 1 µg/µL in reconstitution buffer (5% (v/v) acetonitrile, 0.1% (v/v) formic acid in Milli-Q water. These reconstituted peptides were diluted 1:10 with reconstitution buffer and analyzed using data-dependent acquisition (DDA).

500 ng of peptides (5 µL of the diluted samples) were injected and separated in trap/elute mode, using a nanoAcquity UPLC system (Waters®, England) with a trapping (nanoAcquity Symmetry ® C18, 5 µm, 180 µm × 20 mm) and an analytical column (nanoAcquity ® BEH C18, 1.7 µm, 75 µm × 250 mm). The outlet of the column was coupled to an Orbitrap Fusion Lumos mass spectrometer (Thermo Fisher Scientific) using the Proxeon nanospray source. Solvent A was water, 0.1% FA, and solvent B was acetonitrile, 0.1% FA. Samples were loaded at constant flow of solvent A at 5 µL/min onto the trap for 6 mins. Peptides were eluted via the analytical column at 0.3 µL/min and introduced in the MS via a Pico-Tip Emitter 360 µm OD × 20 µm ID; 10 µm tip (New Objective). A spray voltage of 2.2 kV was used. During the elution step, the percentage of solvent B increased in a non-linear fashion from 0% to 40% in 40 min. Total run time was 60 min, including cleanup and column re-equilibration. The capillary temperature was set to 300 °C.

Samples were acquired in DDA mode with the following MS conditions. Full MS1 scan resolution: 120k, scan range 375–1500 m/z, AGC target: 2 × 10^5^, max injection time: 50 ms. MS2 scan were acquired in the Ion Trap with Rapid scan rate using the following parameter. MIPS: peptide, intensity threshold: 5 × 10^3^, charge state: 2–7, dynamic exclusion: 60 s, isolation width: 1.4 m/z, activation type: HCD, HCD collision energy: 30%, detector type: ion trap, ion trap scan rate: rapid, AGC target: 2 × 10^3^, max injection time: 300 ms, cycletime: 3 s. RF ion funnel was set to 30%. MS1 data were acquired in profile mode and MS2 data were acquired in centroid mode. For data acquisition and processing of the raw data Xcalibur 4.0 (Thermo Scientific) and Tune version 2.1 were employed.

DDA data were searched with MaxQuant 1.5.6.537 [38] against the UniProt human database (database release 2016_01, 20186 entries) using the Andromeda search engine [39]. A list of common contaminants was appended to the database search. The following settings were used: fixed modification was carbamido-methyl (C); variable modifications were oxidation (M) and acetyl (protein N-term), enzyme was Trypsin/P; max. missed cleavages were set to 2; first search peptide tolerance was set to 20 ppm and main search peptide tolerance to 4.5 ppm; match between runs was enabled. All other settings were set to default. PSM and protein hits were filtered at a false discovery rate of 1% using a target-decoy strategy [40]. The iBAQ values per protein (from the proteinGroups.txt output of MaxQuant) were used for further analysis.

### Chromatin immunoprecipitation, RNA extraction, and reverse transcription semi-quantitative real-time PCR (RT-qPCR)

ChIP was performed with the SimpleChIP Kit (Cell Signaling Technology, Canvers, MA, USA) following the manufacturer's instructions. 3 µg of p53 (kind gift from Dr. Bernhard Schlott [41]) antibody was used per IP. Sonication was performed on a Bioruptor Plus (Diagenode, Seraing, Belgium). ChIP-qPCR was performed with a Quantstudio 5 (Thermo Fisher Scientific) using Power SYBR Green MasterMix (Thermo Fisher Scientific) following the manufacturer protocol.

Total cellular RNA was extracted using the RNeasy Plus Mini Kit (Qiagen, Hilden, Germany) following the manufacturer's protocol. One-step reverse transcription and real-time PCR were performed with a Quantstudio 5 using Power SYBR Green RNA-to-CT 1-Step Kit (Thermo Fisher Scientific) following the manufacturer protocol. We used *ACTR10* as a suitable control gene that we identified previously to be unaffected by p53 but expressed across 20 gene expression profiling datasets [6]. Generally, two or three biological replicates with three technical replicates each were used. Given the nature of the technical setup, a few individual data points were erroneous and thus excluded.

Primer sequences are listed in Supplementary Table 1.

### ChIP-seq data

Publicly available RFX7 and p53 ChIP-seq data were visualized through www.targetgenereg.org [33].

### RNA-seq and analysis

Cellular RNA was obtained as described above in biological triplicates. Quality check, polyA+ library preparation, sequencing using an Illumina NovaSeq 6000, read pre-processing, alignment to the human reference genome hg38, and exon level quantification was done as described previously [6].

### Clonogenic assay

HCT116 cells were transfected with 10 nM of respective siRNAs using RNAiMAX. The next day, the transfected cells were seeded six-well plates (50,000 cells per well) containing 2 ml of culture media. After 24 h transfection, cells were challenged with either DMSO or treated with different concentrations of Doxorubicin (0.05, 0.075, 0.1, 0.15, and 0.2 μM) for 24 h. All plates were then recovered in drug-free media and growth continued for another 5 days. After 5 days of recovery, cells were stained with crystal violet containing glutaraldehyde solution and briefly rinsed with plain water.

### Statistics

ChIP and RT-qPCR data were analyzed using a two-sided unpaired *t*-test. Mean Z-scores were compared using a two-sided paired t-test. Violin plots display the median. Bar graphs display mean and standard deviation. *, **, ***, and n.s. indicate *p*-values < 0.05, <0.01, <0.001, and >0.05, respectively. The number of replicates is indicated in each Fig. legend. The experiments were not randomized and investigators were not blinded to allocation during experiments.

### Supplementary information

Supplementary Legends

Supplementary Figure1

Supplementary Figure2

Supplementary Table 1

## Data Availability

The mass spectrometry proteomics data have been deposited to the ProteomeXchange Consortium via the PRIDE [42] partner repository with the dataset identifier PXD020932. RNA sequencing data is accessible through GEO [43] series accession numbers GSE162161 and GSE162162.
