# Supplementary material for: p53 target ANKRA2 cooperates with RFX7 to regulate tumor suppressor genes
Source: Cell Death Discov. 2024 Aug 24;10:376. doi: 10.1038/s41420-024-02149-2 (PMC11344851; doi:10.1038/s41420-024-02149-2)
Supplement: Supplementary file 1 — Supplementary Legends [file 41420_2024_2149_MOESM1_ESM.docx]

**Supplementary Figure 1.**

**(a)** UCSC genome browser images displaying RFX7 ChIP-seq signals and predicted X-boxes at the MXD4 gene locus. Taken from Coronel et al., 2021 Nucl. Acids Res. **(b)** Western blot data from DNA affinity purification eluates using biotinylated MXD4 wild-type (wt) and X-box deleted (Xbox) promoter probes with eluates from DMSO and Nutlin-3a-treated U2OS cells. NF-YA binding served as a loading control. Uncropped western blot images are shown in Supplementary Figure 2.

**Supplementary Figure 2.**

**(a)** Uncropped western blot images corresponding to Figure 1f. **(b)** Uncropped western blot images corresponding to Supplementary Figure 2.
