# Supplementary figures and images for: p53 target ANKRA2 cooperates with RFX7 to regulate tumor suppressor genes
Source: Cell Death Discov. 2024 Aug 24;10:376. doi: 10.1038/s41420-024-02149-2 (PMC11344851; doi:10.1038/s41420-024-02149-2)

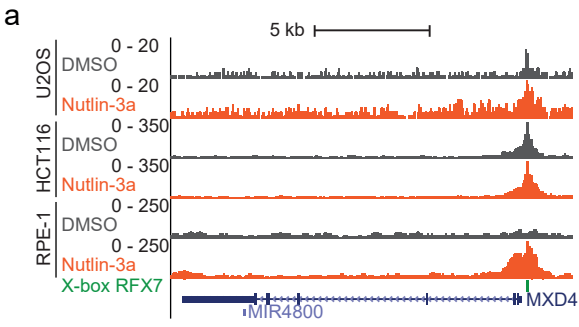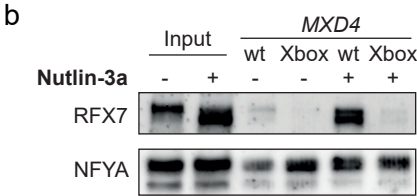

Supplement: Supplementary file 2 — Supplementary Figure1 [file 41420_2024_2149_MOESM2_ESM.pdf]

a

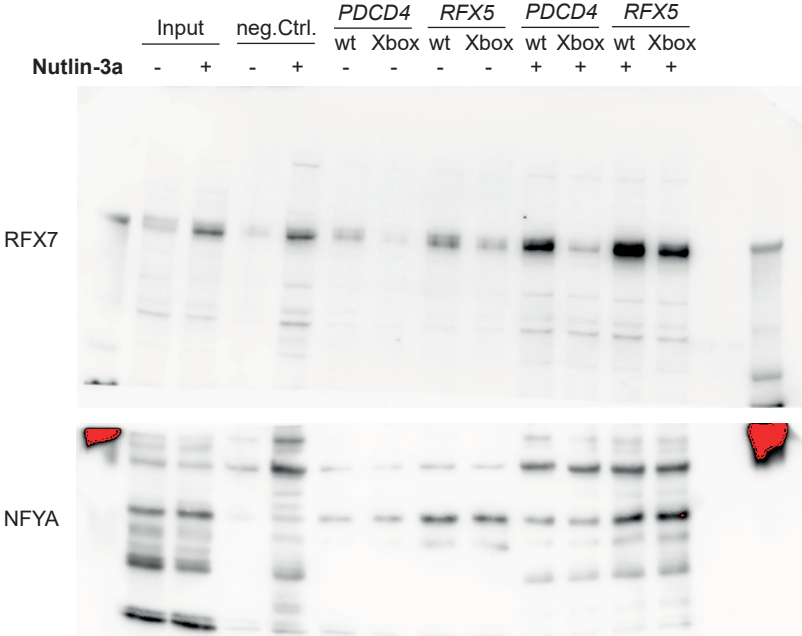

b

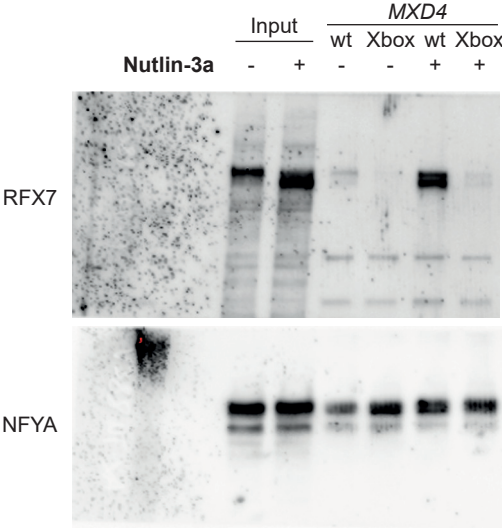

Supplement: Supplementary file 3 — Supplementary Figure2 [file 41420_2024_2149_MOESM3_ESM.pdf]
